# Supplementary material for: Multi‐dataset identification of innovative feature genes and molecular mechanisms in keratoconus
Source: J Cell Mol Med. 2024 Sep 19;28(18):e70079. doi: 10.1111/jcmm.70079 (PMC11412914; doi:10.1111/jcmm.70079)
Supplement: Supplementary file 2 — Material S2. [file JCMM-28-e70079-s002.zip › 7.GSEA analysis/2.ATOH7/KEGG_PORPHYRIN_AND_CHLOROPHYLL_METABOLISM.html]

Details for gene set KEGG\_PORPHYRIN\_AND\_CHLOROPHYLL\_METABOLISM[GSEA]

|  || Dataset | ATOH7.group.cls#h\_versus\_l |
| Phenotype | group.cls#h\_versus\_l |
| Upregulated in class | l |
| GeneSet | KEGG\_PORPHYRIN\_AND\_CHLOROPHYLL\_METABOLISM |
| Enrichment Score (ES) | -0.35397524 |
| Normalized Enrichment Score (NES) | -1.1345707 |
| Nominal p-value | 0.32190475 |
| FDR q-value | 1.0 |
| FWER p-Value | 0.994 |
Table: GSEA Results Summary

  

Fig 1: Enrichment plot: KEGG\_PORPHYRIN\_AND\_CHLOROPHYLL\_METABOLISM      
 Profile of the Running ES Score & Positions of GeneSet Members on the Rank Ordered List

  

| SYMBOL | TITLE | RANK IN GENE LIST | RANK METRIC SCORE | RUNNING ES | CORE ENRICHMENT || 1 | UGT2B7 | na | 1726 | 0.367 | 0.0493 | No |
| 2 | CP | na | 2649 | 0.301 | 0.1040 | No |
| 3 | UGT2B15 | na | 6980 | 0.144 | 0.0180 | No |
| 4 | UGT2A1 | na | 8781 | 0.103 | -0.0061 | No |
| 5 | ALAS1 | na | 8969 | 0.100 | 0.0154 | No |
| 6 | COX10 | na | 10040 | 0.081 | 0.0064 | No |
| 7 | FTMT | na | 10797 | 0.069 | 0.0032 | No |
| 8 | COX15 | na | 11608 | 0.057 | -0.0046 | No |
| 9 | ALAS2 | na | 12022 | 0.052 | -0.0023 | No |
| 10 | HCCS | na | 12425 | 0.047 | -0.0012 | No |
| 11 | UROS | na | 13108 | 0.039 | -0.0102 | No |
| 12 | UGT2B11 | na | 13663 | 0.034 | -0.0171 | No |
| 13 | BLVRA | na | 14270 | 0.028 | -0.0270 | No |
| 14 | GUSB | na | 16038 | 0.013 | -0.0746 | No |
| 15 | BLVRB | na | 16460 | 0.009 | -0.0843 | No |
| 16 | UROD | na | 16666 | 0.007 | -0.0882 | No |
| 17 | UGT2A3 | na | 16675 | 0.007 | -0.0864 | No |
| 18 | FTH1 | na | 16695 | 0.007 | -0.0850 | No |
| 19 | HMOX2 | na | 16765 | 0.007 | -0.0852 | No |
| 20 | EARS2 | na | 17287 | 0.002 | -0.0997 | No |
| 21 | UGT2B10 | na | 19029 | -0.013 | -0.1463 | No |
| 22 | HMBS | na | 19773 | -0.020 | -0.1624 | No |
| 23 | FECH | na | 21933 | -0.042 | -0.2133 | No |
| 24 | CPOX | na | 22225 | -0.045 | -0.2094 | No |
| 25 | PPOX | na | 23139 | -0.057 | -0.2205 | No |
| 26 | HMOX1 | na | 26249 | -0.110 | -0.2805 | No |
| 27 | MMAB | na | 28800 | -0.173 | -0.3073 | Yes |
| 28 | ALAD | na | 28808 | -0.173 | -0.2607 | Yes |
| 29 | UGT2B4 | na | 30951 | -0.245 | -0.2562 | Yes |
| 30 | UGT1A6 | na | 33234 | -0.388 | -0.2173 | Yes |
| 31 | UGT2B28 | na | 33254 | -0.390 | -0.1124 | Yes |
| 32 | UGT1A8 | na | 34302 | -0.573 | 0.0123 | Yes |
Table: GSEA details [plain text format]

  

Fig 2: KEGG\_PORPHYRIN\_AND\_CHLOROPHYLL\_METABOLISM      
 Blue-Pink O' Gram in the Space of the Analyzed GeneSet

  

Fig 3: KEGG\_PORPHYRIN\_AND\_CHLOROPHYLL\_METABOLISM: Random ES distribution      
 Gene set null distribution of ES for **KEGG\_PORPHYRIN\_AND\_CHLOROPHYLL\_METABOLISM**
